# Pilot prospective crossover study assessing the role of bile acid malabsorption in the causation and treatment of major low anterior resection syndrome: randomized clinical trial

**DOI:** 10.1093/bjsopen/zrag141

**Published:** 2026-11-13

**Authors:** John C Woodfield, Kari A Clifford, Rohan Thomas, Simon J G Richards, Anna Kate Fowler, Mohammad K Alquassab, Chrissie Roodt, Mark W Thompson-Fawcett

**Affiliations:** Department of Surgery and Critical Care, Dunedin School of Medicine, University of Otago, Dunedin, New Zealand; Department of Surgery and Critical Care, Dunedin School of Medicine, University of Otago, Dunedin, New Zealand; Department of Surgery, Kew Hospital, Invercargill, New Zealand; Department of Surgery and Critical Care, Christchurch School of Medicine, University of Otago, Christchurch, New Zealand; Department of Surgery, Dunedin Hospital, Dunedin, New Zealand; Department of Surgery, Kew Hospital, Invercargill, New Zealand; Department of Surgery, Dunedin Hospital, Dunedin, New Zealand; Department of Surgery and Critical Care, Dunedin School of Medicine, University of Otago, Dunedin, New Zealand

**Keywords:** colorectal surgery, LARS, quality of life, colesevelam

## Abstract

**Background:**

Major low anterior resection syndrome (LARS) affects approximately 60% of rectal cancer survivors, causing urgency and decreased bowel control, reducing quality of life. Bile acid malabsorption (BAM) is an under-recognized contributor whose prevalence and treatment efficacy remain unclear. This proof-of-concept study evaluated the effectiveness of colesevelam for treating LARS and described the prevalence of BAM.

**Methods:**

This double-blind randomized placebo-controlled crossover trial was conducted between 2021 and 2024 in New Zealand. Adults aged 18–85 years with major LARS received colesevelam (up to 1.25 g, three times daily) or placebo for 5 weeks, separated by a 6-week washout period. A subset of patients underwent 75-selenium homocholic acid taurine (SeHCAT) testing for BAM. The outcomes of interest included changes in the LARS score, Fecal Incontinence Quality of Life Scale (FIQL), and Short-Form 12 (SF-12) questionnaire, with a composite outcome reflecting clinically important improvements.

**Results:**

Of 80 eligible patients, 49 were randomized. However, 36 patients completed the study after washout and were included in a per-protocol analysis (mean age 71 years; 69% male). In these 36 patients, colesevelam did not significantly improve LARS scores, but did significantly improve the SF-12 mental component summary (median +2.36; 95% confidence interval 0.00 to 10.25; *P* < 0.01) and FIQL depression/self-perception. Eight of 19 patients (42%) had BAM and exhibited significant improvements in SF-12 physical and mental item scores with colesevelam. Using composite criteria, colesevelam produced a 33% response rate, *versus* 8% for placebo, with higher rates in BAM-positive (50%) than BAM-negative (11%) patients.

**Conclusions:**

BAM is a treatable contributor to LARS, present in 40% of tested patients. Although treating all LARS patients with bile acid sequestrants provides limited benefit, SeHCAT testing identifies patients likely to respond. BAM assessment would be beneficial if incorporated into LARS management.

## Teaser

This double-blind randomized crossover trial evaluated whether bile acid malabsorption (BAM) contributes to major low anterior resection syndrome (LARS) and whether treatment with the bile acid sequestrant colesevelam is effective. Among 36 patients with major LARS, colesevelam did not significantly improve overall LARS scores but did improve quality-of-life measures, particularly mental health outcomes, with a greater benefit seen in patients with confirmed BAM, which was present in approximately 40% of those tested. The findings suggest that although empirical bile acid sequestrant therapy has limited benefit. However, targeted treatment guided by 75-selenium homocholic acid taurine testing can identify LARS patients more likely to respond and should be incorporated into management pathways.

## Introduction

The success of multimodal treatment of rectal cancer has resulted in the quality of survivorship becoming increasingly important. Approximately 60% of patients may have major and ongoing functional problems with an associated decrease in quality of life (QoL) after anterior resection^1–4^. Urgency, poor control of flatus and stool, frequency, and incomplete emptying have been incorporated into a quantified scoring system known as the low anterior resection syndrome (LARS) score^1^. The causes of LARS are multifactorial, including the removal of the rectum, autonomic denervation^5^, pelvic radiation disease^2^, chemotherapy, and damage caused by endoanal instrumentation^6^.

Small bowel pathologies that cause frequent, loose motions are another cause of LARS. Bile acid malabsorption (BAM) is one such small bowel pathology. As part of the enterohepatic circulation, approximately 95% of bile is absorbed in the ileum and returns to the liver. Malabsorption in the ileum increases bile acids in the colon. This induces sodium, water, and mucous secretion, increases colonic motility, and may damage the mucosa, increasing mucosal permeability^7^. A likely cause of BAM after multimodal treatment of rectal cancer is radiation damage to the terminal ileum. In the setting of pelvic radiotherapy for malignancy, BAM is believed to contribute to 35–72% of diarrhoea caused by radiation enteritis^8^. Other contributing causes include a preoperative predisposition to BAM, and, in this setting, surgery involving the ileum, small intestinal bacterial overgrowth, and villous atrophy related to chemotherapy or a prolonged loop ileostomy may be important. It is also estimated that 1% of the general population has BAM^9^. Although BAM is occasionally considered as a cause of LARS^10–12^, it is not known how frequently it is a contributing factor or the main cause of LARS.

The standard for diagnosis of BAM is the 75-selenium homocholic acid taurine (SeHCAT) test. A selenium-labelled bile acid capsule is taken orally, with gamma camera scans on days 0 and 7 providing decay-corrected retention values. Day 7 retention below 10% confirms BAM with a sensitivity and specificity of 87% and 93%, respectively^13,14^. BAM is managed by binding bile acid with sequestrants to prevent irritation of the colonic mucosa^15^. Although effective in treating BAM in approximately 70% of patients^8^, the powdered forms of cholestyramine and colestipol are poorly tolerated due to taste, texture, and symptoms of nausea, resulting in poor long-term compliance^9,16^. Colesevelam which is six-fold more potent than other bile acid sequestrants can be administered as a capsule. The compound has no taste; thus, a placebo capsule can be manufactured, making this a suitable bile acid sequestrant to use in a blinded randomized clinical trial. This study was designed as a proof-of-concept study to determine the contribution of BAM to LARS and the efficacy of treating LARS with a bile acid sequestrant.

## Methods

### Design and participants

This was a prospective double-blinded randomized placebo-controlled pilot study conducted between October 2021 and March 2024, with a two-period crossover design. Each treatment period was for 5 weeks, with a 6-week washout between interventions to account for first-order carryover effects.

Included participants had an anterior resection for rectal cancer after 1999, were aged ≥ 18 years, and had bowel continuity re-established. They had been treated in the South Island of New Zealand and had major LARS defined by a LARS score > 30, with a tendency towards frequent loose motions. Exclusion criteria included age over 85 years, an inability to answer questionnaires, or an identified allergy or intolerance to colesevelam.

Clinical data were collected from electronic medical records and the Otago Clinical Audit, which documents all operations and their complications. The information collected included demographics, operative details, other cancer treatments, histopathology, SeHCAT results, questionnaire outcomes, and secondary endpoints.

The study was conducted in full conformity with the Declaration of Helsinki^17^ and with the International Council for Harmonisation of Technical Requirements for Pharmaceuticals for Human Use Guidelines for Good Clinical Practice (CPMP/ICH/135/95)^18^. The trial was registered with the Australian New Zealand Clinical Trials Registry (ACTRN12620000755932) and was approved by the New Zealand Health and Disability Ethics Committee (HDEC 19/CEN/209). The funders and the sponsors had no oversight of this trial.

### Interventions

Participants were randomized by a researcher (K.A.C.) in a 1 : 1 ratio to treatment sequence (colesevelam-first *versus* placebo-first) using a computer-generated random number sequence without blocking, using sequentially arranged opaque envelopes.

Colesevelam was presented as 312-mg capsules. The placebo, an identical-looking capsule, contained an inert excipient (dextrose). Participants and assessors were blinded to the treatment. To manage potential constipation, participants started with one capsule three times a day for three days (312 mg, three times a day), increasing to two capsules three times a day and then to four capsules three times a day (1.25 g three times a day) after the first week. Trial medications were taken at least 2 hours before or after other medications. The SeHCAT test was performed on a subset of participants for financial reasons. Because the test required two visits to the Nuclear Medicine Department in Dunedin, this was offered to all participants living in Dunedin until funds were exhausted.

### Outcomes of interest

The primary objective of this study was to determine the efficacy of treating major LARS with the bile acid sequestrant colesevelam. Three questionnaires were used. Efficacy was defined as a change in LARS score^1^ of −5. This is a validated symptom severity score with a five-point improvement being recognized as the minimal clinically important difference (MCID)^2,3^. Other symptoms and consequences of LARS were measured using two QoL evaluations: the FIQL, which has been used in LARS research^4–6^ and assesses the impact of bowel function in the domains of lifestyle, behaviour, self-perception, and embarrassment; and the 12-Item Short-Form Survey (SF-12)^19^, which provides general physical component summary (PCS) and mental component summary (MCS) scores^20–22^.

The secondary objective of the study was to use SeHCAT testing to quantify the contribution of BAM to LARS and to document the association between BAM severity and response to colesevelam. BAM was diagnosed when retention of the SeHCAT radiological marker on day 7 was < 10%. Individual questionnaire endpoints and a composite outcome were used to identify a response to colesevelam in participants with and without BAM. Data collection included a stool diary documenting the symptoms of LARS, the use of additional medicines, and adverse events. Participants identified which treatment they found more helpful and scored their response on a five-point Likert scale. The composite outcome for overall response was based on MCIDs in the LARS score, FIQL, and SF-12 of −5, 1.9, and 5, respectively, and in patients rating the active treatment 4 or 5 on the Likert scale. A positive composite outcome required at least three of the four outcomes to improve, two by the described MCID^8–10^. No individual score could deteriorate by the MCID and the response to colesevelam had to be considerably better than to placebo.

Participants were contacted eight times over the duration of the study (weekly for the first 4 weeks of each treatment) to check for adverse events and side effects. These were assessed for severity and degree of causality using Common Terminology Criteria for Adverse Events Version 4^23^.

### Statistical analysis

Sample size was estimated using PASS (Power analysis and sample size software, NCSS LLC). Standard deviations were determined from our Dunedin Departments prospective LARS database and from LARS scores in similar populations^24^. For an inequality test on data with a two-period crossover design, a sample size of 36 achieves 80% power at a 5% significance level when the absolute difference in LARS score between the two treatment means is 5 and the within-subject standard deviation (s.d.) is 10. This study aimed to recruit 45 participants to allow for attrition. Patient characteristics are presented using descriptive statistics. For the primary and secondary endpoints, changes in LARS, FIQL and SF-12 scores were compared using the Wilcoxon rank-sum test. All analyses were completed on a per-protocol basis.

To assess the effect of the treatment on all participants, a mixed-effects linear model regression analysis was performed for the primary outcomes. This accounted for potential period and participant effects. Mean imputation was used for any missing data with a sensitivity test to assess whether imputation affected the direction of association. Analyses in the subset of participants undergoing SeHCAT assessment included regression analysis accounting for the drug effect (colesevelam or placebo), BAM status effect, and the interaction between the drug and BAM status. The relationship between the SeHCAT test and response, as measured with the composite outcomes in three severity categories of BAM (< 5%, 5–10% and > 10%^25^) was assessed with the Cochran–Armitage trend test. All statistical analyses were performed using R version 4.4.0 (R Foundation for Statistical Computing, Vienna, Austria), with *P* < 0.05 considered significant.

## Results

Of 289 potential participants screened, 80 were identified who met eligibility criteria. Reasons for not enrolling are outlined in *Fig. 1*, including seven patients (8.7% of those identified) with constipation or an irregular bowel habit with intermittent constipation. Of the 49 patients who were randomized, seven withdrew while taking colesevelam and six withdrew while taking placebo. The reasons for withdrawal included constipation (three colesevelam, one placebo), unavailability (three), felt no response (two), not stated (two), symptoms settling before treatment (one), and medication error (one). Complete data for analysis were available for 36 patients.

**Figure 1:**
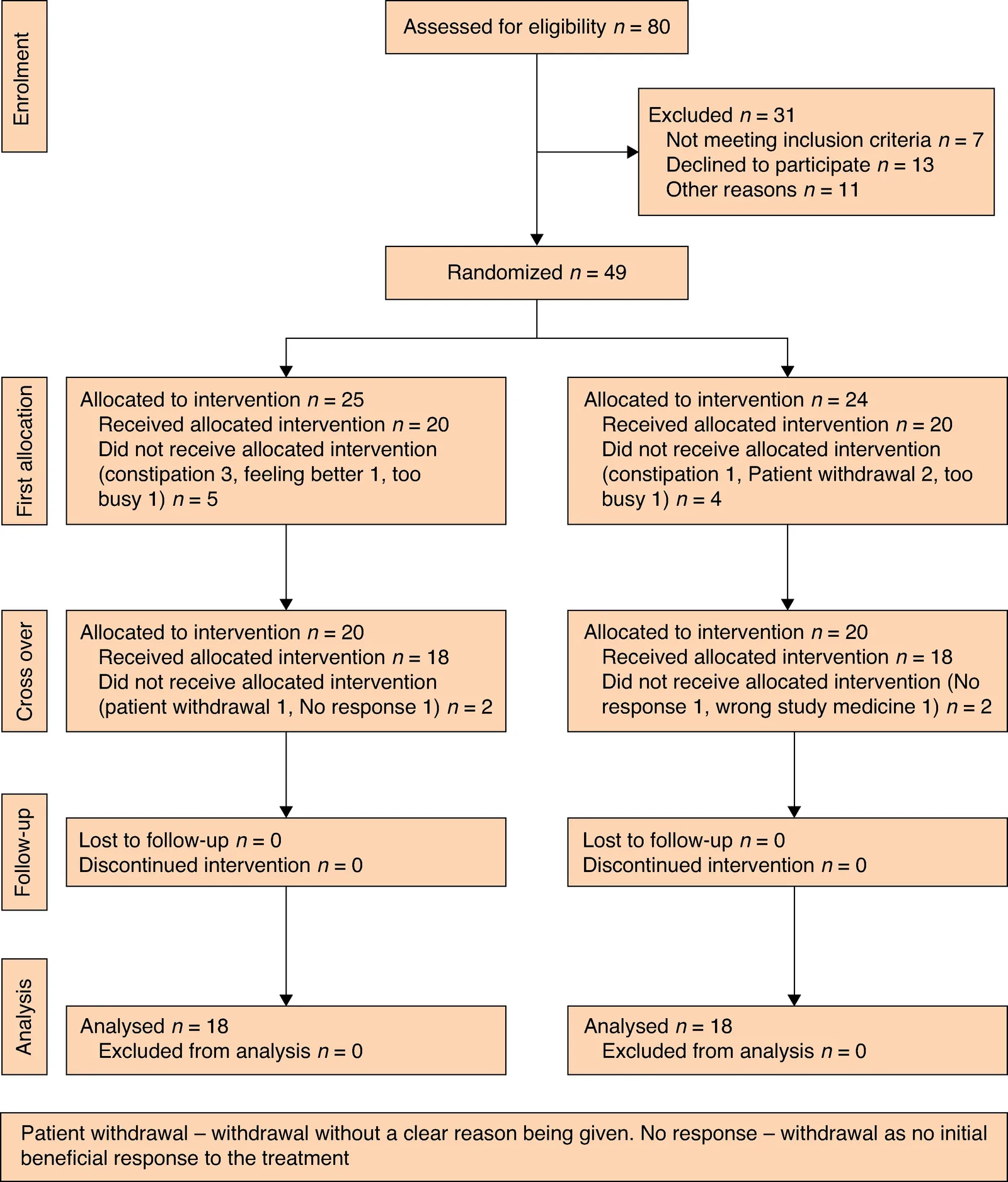
Study flow diagram

Of these 36 patients (*Table 1*), 69.4% were male. The median age at surgery was 64 (interquartile range (i.q.r.) 46–83) years, and at that at enrolment was 71 (i.q.r. 50–85) years. Ethnically, Europeans comprised 97% of patients. Therapy for rectal cancer included radiotherapy (58%), chemotherapy (64%), and neither (25%). Participants had a median of 4.2 (i.q.r. 2.27–5.88) years between index surgery and enrolment. Thirty-three per cent of surgeries were for stage IIIB pathology. The median height of the tumour from the anal verge was 9 cm. Twenty-eight participants (78%) received an ileostomy, all of which were reversed at the time of enrolment.

**Table 1:** Participant characteristics and history of treatment

	Colesevelam to placebo (*n* = 18)	Placebo to colesevelam (*n* = 18)	Total (*n* = 36)	SeHCAT subset (*n* = 16)
**Sex**				
Male	6 (67%)	13 (72%)	25 (69%)	12 (75%)
Female	12 (33%)	5 (28%)	11 (31%)	4 (25%)
**Age (years), median (i.q.r.)**				
At study enrolment	66.5 (60.0, 73.0)	74.5 (65.5, 77.0)	71 (61.8, 76.0)	72.5 (60.8, 76.3)
At anterior resection	58.5 (54.3, 67.5)	67 (60.0, 72.8)	64 (56.0, 70.3)	66 (56.0, 70.0)
**Ethnicity**				
European	17 (94%)	18 (100%)	35 (97%)	15 (94%)
Asian	2 (11%)	0 (0%)	2 (6%)	2 (13%)
Māori	1 (6%)	0 (0%)	1 (3%)	0 (0%)
Other*	0 (0%)	1 (6%)	1 (6%)	0 (0%)
Prior ileostomy and reversal	15 (83%)	13 (72%)	28 (78%)	15 (94%)
Radiotherapy	11 (61%)	10 (56%)	21 (58%)	9 (56%)
Chemotherapy	10 (56%)	13 (72%)	23 (64%)	8 (50%)
No radiotherapy or chemotherapy	5 (28%)	4 (22%)	9 (25%)	4 (25%)
Tumour location from anal verge (cm), median (i.q.r.)	9 (7.0, 9.8)	9 (7.0, 10.8)	9 (7.0, 10.0)	8 (7.0, 9.0)
**Stage†**				
I	3 (17%)	6 (33%)	9 (25%)	2 (13%)
IIA	7 (39%)	2 (11%)	9 (25%)	5 (31%)
IIB	1 (6%)	1 (6%)	2 (6%)	1 (6%)
IIIA	1 (6%)	3 (17%)	4 (11%)	1 (6%)
IIIB	6 (33%)	6 (33%)	12 (33%)	7 (44%)

Values are *n* (%) unless otherwise stated. *Other ethnicity implies not European, Māori, Pacific, Asian, or Middle Eastern/Latin American/African. †Staging according to the American Joint Committee on Cancer guidelines (8th edition)^26^. SeHCAT, 75-Selenium homocholic acid taurine; i.q.r., interquartile range.

SeHCAT testing was completed in 19 participants. Of these, 17 had response data to colesevelam and 16 completed the study (*Fig. S1*). The demographics and treatment for those with SeHCAT tests were similar to the overall patient group, apart from more of the SeHCAT subset of patients having had an ileostomy (94%).

### Efficacy of treating major LARS

Analysis of treatment of all patients with colesevelam showed no significant change in LARS score, although this improved by −5 in 25% of patients (*Table 2*).

**Table 2:** Effect of colesevelam on LARS and quality of life scores in 36 patients†

	Colesevelam	Placebo	P*
Change in LARS score, median (i.q.r.)	0.00 (−5.00, 0.00)	0.00 (−3.00, 0.00)	0.645
**Change in FIQL, median (i.q.r.)**			
Lifestyle	0.00 (−0.10, 0.30)	0.00 (−0.10, 0.22)	0.438
Coping/behaviour	0.28 (−0.09, 0.58)	0.11 (0.00, 0.29)	0.194
Depression/self-perception	0.05 (−0.03, 0.57)	0.00 (−0.14, 0.29)	0.018
Embarrassment	0.33 (0.00, 0.67)	0.00 (0.00, 0.33)	0.575
**Change in SF-12, median (i.q.r.)**			
PCS	−0.42 (−4.81, 3.68)	0.00 (−2.36, 2.21)	0.892
MCS	3.46 (0.00, 10.25)	0.00 (−1.24, 2.25)	< 0.001

^†^Approximately 2% of scores are imputed. Imputation did not change the trends or significance between colesevelam and placebo. LARS, low anterior resection syndrome; i.q.r., interquartile range; FIQL, Fecal Incontinence Quality of Life Scale; SF-12, 12-Item Short Form Survey; PCS, physical component summary; MCS, mental component summary. *Linear mixed-effect models with participant as a random effect were fitted to estimate *P* values.

For the FIQL, an improvement was noted in three of four domains, being significant for depression/self perception (*P* = 0.02), with 25% of patients having an improvement of 40% over baseline (*Fig. S2*). For the SF-12 MCS score, there was a median improvement of 3.5 points compared with placebo (*P* < 0.001; *Fig. S3*) with greater differences in coping, perception, and psychological scores than in physical scores.

### Contribution of BAM to LARS

BAM was confirmed in eight of 19 patients (42%). Full analysis of the response to treatment with colesevelam and placebo was available for 16 patients who had a SeHCAT test (*Table 3*).

**Table 3:** Effects of treatment on LARS and quality of life scores stratified by BAM status

	Those with BAM (*n* = 8)		Those without BAM (*n* = 8)	
	Colesevelam	Placebo	Colesevelam	Placebo
Change in LARS score, median (i.q.r.)	−2.50 (−6.50, 0.00)	0.00 (0.00, 0.00)	0.00 (−0.75, 0.25)	0.00 (−1.50, 0.00)
**Change in FIQL, median (i.q.r.)**				
Lifestyle	0.20 (−0.02, 0.50)	0.00 (−0.02, 0.15)	0.00 (−0.07, 0.07)	0.00 (−0.2, 0.12)
Coping/behaviour	0.56 (0.14, 0.67)	0.17 (0.00, 0.42)	0.28 (0.08, 0.61)	0.17 (0.08, 0.25)
Depression	0.36 (−0.04, 0.71)	0.00 (0.00, 0.21)	0.00 (−0.36, 0.43)	0.07 (−0.11, 0.21)
Embarrassment	0.33 (0.25, 0.67)	0.00 (0.00, 0.25)	0.00 (−0.08, 0.08)	0.00 (0.00, 0.33)
**Change in SF-12, median (i.q.r.)**				
PCS	2.25 (−0.94, 9.67)	−0.06 (−5.83, 0.29)	−0.56 (−4.67, 0.00)	−0.06 (−2.61, 2.21)
MCS	7.43 (2.63, 14.51)	1.87 (0.51, 2.66)	2.68 (0.00, 9.26)	−0.10 (−1.14, 1.35)

LARS, low anterior resection syndrome; BAM, bile acid malabsorption; i.q.r., interquartile range; FIQL, Fecal Incontinence Quality of Life Scale; SF-12, 12-Item Short-Form Survey; PCS, physical component summary; MCS, mental component summary.

There was an improvement in all questionnaire measurements after colesevelam therapy in those with BAM (*Figs S4* and *S5*). Limited numbers contributed to no significant differences in LARS scores or FIQL scale domains. There were significant improvements in the SF-12 PCS and MCS scores (*Figs S6* and *S7*). An improvement in the PCS score was significantly associated with colesevelam (*P* = 0.029) and BAM status (*P* = 0.010). The greatest PCS improvement occurred when colesevelam was administered to patients with BAM (*P* = 0.016). For the MCS score, colesevelam independently produced a significant improvement (*P* = 0.038). Because colesevelam benefited both BAM and non-BAM groups, BAM status was not significantly associated with the effect of treatment on MCS (*P* = 0.710).

### Overall clinical response

The composite outcome was used as an overall measurement of clinical response (*Table 4* and *Tables S1–S3*). Twelve of 36 participants (33%) responded to treatment with colesevelam. Three of 36 participants (8%) responded to treatment with placebo. The response to colesevelam when accounting for BAM severity (17 participants) was three of five participants with a retention of < 5%, one of three participants with retention between 5% and 10% and one of nine participants with retention > 10% (*P* = 0.053). The diary for the patient who improved with SeHCAT retention > 10% documented that the constipating effects of colesevelam helped by reducing the frequency of loose motions. In terms of pathophysiology, of the 12 patients responding to treatment for BAM, nine (75%) had previously received radiotherapy, eight (67%) had received chemotherapy, six (50%) had received both, and two (16%) had received neither (*Table S4*). Adverse events reflected the symptoms of LARS and were similarly reported in both treatment periods (*Table S5*).

**Table 4:** Effect of colesevelam treatment based on knowledge of BAM status

Response to treatment	Likelihood of response (%)
Confirmed BAM with SeHCAT testing	4 of 8 (50%)
Not tested for BAM	
For those who completed the study	12 of 36 (33%)
For those who were recruited to the study	12 of 49 (24%)
Confirmed not to have BAM with SeHCAT testing	1 of 9 (11%)

BAM, bile acid malabsorption; SeHCAT, 75-selenium homocholic acid taurine.

## Discussion

The primary objective of this study was to determine the overall effectiveness of colesevelam in the treatment of patients with major LARS with symptoms of loose or frequent bowel motions. This addresses the clinical scenario of trialling therapy for a patient with LARS with no access to SeHCAT testing. Using colesevelam in this setting did not improve the LARS score. The improvement of −5 in one-quarter of patients is consistent with BAM being one of many aetiologies for LARS. There was an improvement in the SF-12 MCS score and FIQL depression/perception scale. Because the LARS score focuses on physical rather than mental or psychological aspects of LARS, this result is consistent with an improvement in physical symptoms in a subset of participants being sufficient to significantly improve some of the psychological consequences of LARS.

A second objective of the study was to estimate the prevalence of BAM in major LARS. This has not been previously described, partly because of limited use of the SeHCAT test. Of 19 sequentially recruited patients from the Dunedin region in New Zealand who underwent SeHCAT testing, 42% had BAM, with a SeHCAT retention of < 10%. Although this sample size is small, the findings suggest that BAM is a sizeable contributor to the pathophysiology and presentation of LARS.

This analysis, limited by its sample size, also showed that participants with BAM had trends favouring colesevelam in all primary outcome measures, including significant improvements in the SF-12 PCS and MCS scores. A separate analysis of 17 participants with SeHCAT scores and clinical response data to colesevelam demonstrated borderline significance between the severity of BAM and a clinical response. When accounting for BAM, there were improvements in LARS in 50% (4 of 8) of patients with BAM and in 11% (1 of 9) of patients without BAM. These limited observations indicate that BAM status helps predict the likelihood of successful treatment. The results suggest that BAM should be included in treatment algorithms for managing LARS. However, why did only 50% of patients with BAM respond? There are two contributing reasons. First, patients may have more than one cause of LARS. If BAM is the main cause, then treating this is likely to be beneficial. If there are other important contributors to LARS, then both BAM and other causes need to be addressed to improve symptoms. Second, bile acid sequestrants do not successfully treat all patients with BAM^27^. Alternative treatments, for example looking at feedback pathways to the liver to reduce bile acid production, are being explored^28^.

This study used colesevelam capsules, which are easier to take than bile acid sequestrant powders. Colesevelam has a range of common side effects, including constipation^29–31^. Patients were recruited who had a tendency towards frequent loose motions, excluding seven of 80 identified participants with difficulty emptying. Constipation was managed by starting with a low dose of colesevelam and increasing this over 2 weeks. Limited evidence suggests that the effective dose of colesevelam for treating BAM can be as low as 1.25 g/day^32^ and as high as 3.75 g/day, although most sources would recommend the higher dose^32–34^. The results suggest that the dose of colesevelam should be personalized to ensure that it is high enough to be effective while minimizing side effects.

In this report, a composite outcome rather than the LARS score was used to assess the overall response of LARS to colesevelam. The composite outcome captured a wide range of symptoms and consequences consistent with the recent consensus statement definition of LARS^35^. In contrast, the LARS score focuses on the physical symptoms of LARS. The structure of the LARS score can also create anomalies; for example, having the same score for a patient with fragmentation daily or once a week. Sometimes the impact on QoL is overestimated in patients with urgency and incontinence and underestimated in patients with severe defaecatory disorders^36^. Discordance between the LARS score and the patient's self-assessment of their bowel function and QoL has also been reported^37^. Changes in LARS score were sometimes contradictory to details reported in patients’ diaries and there was poor correlation between the LARS score and other questionnaires (*Table S6*). The results of the present study support those of Ribas *et al*.^36^, who stated:

In our opinion, due to the complexity of the syndrome, the LARS score may be a useful tool for screening, but it is not subtle enough to properly assess bowel dysfunction after rectal cancer treatment, or the outcomes of any therapeutic procedure.

This study illustrates the need for a more comprehensive scoring system for LARS. The composite outcome was drawn from the data that were available. Further research is needed to develop a scoring system for LARS that will provide a better holistic view of a patient's disease and response to treatment.

Weaknesses with this study, including the use of an unvalidated composite outcome for documenting overall clinical response and a limited sample size, have been mentioned. The small SeHCAT subgroup means reported response rates for treating LARS based on BAM status are estimations. If all patients had a SeHCAT test, the results would have been more robust. Additional studies with a larger sample size of patients with known BAM status are needed to confirm these findings. The recruitment of 49 of 80 eligible contacted patients may have also introduced unidentified bias into the study. This study's strength is that it is a prospective double-blind randomized placebo-controlled two-period crossover trial. This decreases confounding bias, researcher bias, and performance bias, and enables an assessment of the placebo effect on bowel symptoms and clinical outcomes^38,39^. Because three patients in the placebo group and 12 in the colesevelam group responded to treatment, it is likely that one-quarter of the response is related to the placebo effect.

This pilot study has addressed a knowledge gap in the assessment and treatment of LARS. The estimated prevalence of BAM in major LARS with loose or frequent motions was approximately 40%, being more important than previously appreciated. Investigating for BAM with a SeHCAT test was useful, with approximately 50% of patients with BAM responding to treatment with a bile acid sequestrant. Treatment of all patients without knowing BAM status did not improve the LARS score, but was associated with an overall clinical improvement in one-third of patients. When adjusting for the placebo effect (up to one-quarter of this benefit may be related to the placebo effect) and patient withdrawals, this clinical improvement may be as low as one in five patients. The results suggest a benefit in improving access to SeHCAT testing and including BAM in algorithms for assessing and treating LARS. Further research is required to confirm these observations.

## Supplementary Material

zrag141_Supplementary_Data

## Data Availability

Deidentified study data are available upon reasonable request to the corresponding author.
