# Supplementary material for: Pilot prospective crossover study assessing the role of bile acid malabsorption in the causation and treatment of major low anterior resection syndrome: randomized clinical trial
Source: BJS Open. 2026 Nov 13;10(6):zrag141. doi: 10.1093/bjsopen/zrag141 (PMC13765537; doi:10.1093/bjsopen/zrag141)
Supplement: Supplementary file 1 [file zrag141_supplementary_data.zip › Supplementary_Materials.docx]

**Title**

A pilot prospective cross over study assessing the role of bile acid malabsorption in the causation and treatment of major low anterior resection syndrome

A Randomised clinical trial

**Authors:** John C. Woodfield, PhD^1^; Kari A. Clifford, PhD^1^; Rohan Thomas, MBChB^2^; Simon JG Richards, FRACS^3^; Anna Kate Fowler, PhD^4^; Mohammad K Alquassab, MBChB^2^; Chrissie Roodt, PgDip^4^; Mark W Thompson-Fawcett, FRACS^1^.

^1^ Department of Surgery and Critical Care, Dunedin School of Medicine

^2^ Kew Hospital, Invercargill

^3^ Christchurch Hospital, Christchurch

^4^ Dunedin Hospital, Dunedin

**Corresponding Author:**

John Woodfield

Department of Surgery and Critical Care

Dunedin School of Medicine

University of Otago

John.woodfield@otago.ac.nz

**Supplementary Materials - Index**

| **Supplementary Methods** |  | | |
| --- | --- | --- | --- |
| Participant perspective | *page 2* | | |
| Rationale and definition of composite outcome | *page 2* | | |
| **Supplementary Figures and Tables** |  | | |
| Figure S1: Consort Flow Diagram for participants with SeHCAT tests | *page 4* | |  |
| Figures S2-S7: Effects of colesevelam on quality of life scores | *pages 5-7* | |  |
| Table S1: Participant impressions of bowel symptom changes following therapy with the study medication | *Page 8* | |  |
| Table S2: The number of participants determined to have a response to colesevelam as suggested by each endpoint, and the contribution of each endpoint to the composite outcome. | *Page 8* | |  |
| Table S3: The overall response to colesevelam and placebo compared to a clinically important response for the different individual outcome measures | *Page 8* | |  |
| Table S4: Relationship between Rx and BAM – in the 17 patients with SeHCAT and had colesevelam n(%) |  |  |  |
| Table S5: Number of patients reporting adverse events following therapy n(%) | *Page 8* |  |  |
| Table S6: Spearman rank correlation coefficients between change in LARS score and changes in the FIQL scales and SF-12 scores | *Page 8* |  |  |
|  |  | | |

**Supplementary Methods**

**Participant impression of bowel symptom changes following therapy**

Participants were asked to assess if they found the first or second treatment more helpful in relieving their symptoms, and to quantify the extent of the change on a five-point Likert scale (anchors shown in Table S1). The results are summarised in the context of if the treatment patients found more helpful was or was not aligned with administration of colesevelam.

**Rationale for a composite outcome measurement to assess response to treatment.**

The new consensus definition of LARS looks at eight symptoms and consequences of LARS. Given the limitations of any one individual study endpoint, which looks at some aspects of LARS, but does not consider other important symptoms and consequences, a basic exploration was performed to see if combining the three primary outcome measures (LARS score, FIQL scales, and SF-12 survey) and the secondary endpoint of the patient's overall impression of clinical response, would provide a better overall indication of response to therapy. This was termed a ‘composite outcome.’ The point of using this was not to create a new gold standard, rather, it was a mechanism to allow us to account for and interpret the full range of outcomes patients reported to us.

The composite outcome is a combination of the following four outcomes: the LARS score, SF-12 QoL scores, FIQL scales, and the patient's overall impression (Table S1) of clinical response. To have a response to treatment, at least three of the four outcomes had to be positive, at least two of these by a clinically important margin. A clinically important margin was an improvement in LARS score of -5, FIQL of 1.9, SF-12 of 5 and the patient’s overall impression of clinical response being helpful or very helpful. No individual score could deteriorate by the above clinically important margin, and the response to colesevelam had to be considerably better than to placebo (and vice versa for a placebo response).

The following reasons contributed to this decision:

**1. Consensus statement: The composite score was used as the best overall measurement of clinical response.**

This was because no one score adequately captured the symptoms and consequences of LARS. The composite outcome is therefore more consistent with the recent consensus definition of LARS, which documents eight symptoms and eight consequences of LARS.

**2. The LARS score focuses on physical symptoms**.

The LARS score focuses on primary symptoms and does not engage with psychological and mental, symptoms and consequences of LARS. In our study, in the analysis of all patients, we noted that a small change in the LARS score was associated with significant changes in the FIQL depression/ perception scale and significant changes in the SF-12 MCS score. The LARS score was not capturing these important improvements in patient’s well-being.

**3. The structure of the LARS score can sometimes create anomalies**.

For example, a small change in incontinence to wind from less than once per week to once a week increases the LARS score from four to seven. In contrast, fragmentation within one hour has the same score of 11 if this happens daily episodes or if this happens once a week.

This means that the way the score is weighted can contribute to patients with faecal incontinence having a higher LARS score than their change in QoL suggests, and those with defecatory problems have been documented to have a lower LARS score than their change in QoL suggests.

In this context we noted that sometimes there were large changes in LARS score with minor changes in symptoms, with the changes in LARS score sometimes being at variance with all other measurements of response to treatment. Examples include the LARS score improving when all other measurements suggested no improvement or a deterioration. On other occasions, the LARS score did not change or was slightly worse when all other endpoints showed an important clinical improvement.

**4. Inaccurate reporting of LARS score when compared to the patient’s diary.**

We noticed that patients often scored the LARS score based on their impressions rather than an accurate review of symptoms. This was confirmed when comparing the LARS questionnaire with the patient diary. For example, some patients whose LARS score had improved due to reporting a reduction in frequency of accidents in the LARS questionnaire had no reduction in the frequency of accidents in their diary. A range of similar examples were documented.

**Supplementary Figures and Tables**

**Figure S1 CONSORT Flow diagram for participants undergoing SeHCAT testing**

**Figure S2: The effect of colesevelam on FIQL depression/Self Perception**

**Figure S3. The effect of colesevelam on SF-12 MCS**

**Figure S4. The effect of colesevelam on LARS scores when stratified by BAM status**

An improvement in LARS score is indicated by a reduction in score.

**Figure S5. The effect of colesevelam on FIQL depression / self-perception scores when stratified by BAM status**

**Figure S6. The effect of colesevelam on SF 12 PCS scores when stratified by BAM** **status**

**Figure S7. The effect of colesevelam on SF 12 MCS scores when stratified by BAM status**

**Table S1. Participant impressions of bowel symptom changes following therapy with the study medication^*^**

| **Category** | **Number(%) responding** |
| --- | --- |
|  |  |
| **Worse** | 1 (3) |
| **No Change** | 16 (44) |
| **Slight Change** | 8 (22) |
| **Helpful** | 9 (25) |
| **Very Helpful** | 2 (6) |

^*^ For those rating the medication “Slight change”, “Helpful”, or “very Helpful”, all but one respondent referred to the colesevelam condition. This respondent referred to the placebo condition as “Helpful”. The participant who responded “Worse” referred to the placebo period.

**Table S2. The number of participants determined to have a response to colesevelam as suggested by each endpoint, and the contribution of each endpoint to the composite outcome.**

| Endpoint | Patient impression | FIQL score | SF-12 score | LARS score |
| --- | --- | --- | --- | --- |
| Response | 10 | 10 | 14 | 10 |
| Included in composite score | 8 | 9 | 12 | 7 |

**Table S3 The overall response to colesevelam and placebo compared to a clinically important response for the different individual outcome measures**

|  | Clinical | FIQL | SF-12 | LARS score | Composite |
| --- | --- | --- | --- | --- | --- |
| Colesevelam | 10/36 (28%) | 10/36 (28%) | 14/36 (39%) | 10/36 (28%) | 12/36 (33%) |
| Placebo | 1/36 (3%) | 4/36 (11%) | 6/36 (17%) | 6/36 (17%) | 3/36 (8%) |

**Table S4. Relationship between Rx and BAM – in the 17 patients with SeHCAT and had colesevelam n(%)**

|  | N | Radiotherapy | Chemotherapy | Both | Neither |
| --- | --- | --- | --- | --- | --- |
| Total | 36 of 36 | 21 (58) | 23 (72) | 16 (44) | 9 (25) |
| Response to Rx | 12 of 36 | 9 (75) | 8 (67) | 6 (50) | 2 (16) |
| BAM | 8 of 17 | 4 (50) | 3 (37) | 2 (25) | 3 (37) |
| BAM + Response to Rx | 4 of 8 | 2 (50) | 1 (25) | 0 | 1 (25) |

**Table S5. Number of patients reporting adverse events following therapy n(%)**

| Treatment | Nausea | Vomiting | Constipation | Bloating | Headache |
| --- | --- | --- | --- | --- | --- |
| Colesevelam | 5 (13.8) | 1 (2.7) | 6 (16.6) | 7 (19.4) | 4 (11.1) |
| Placebo | 2 (5.5) | 1 (2.7) | 6 (16.6) | 6 (16.6) | 6 (16.6) |
|  |  |  |  |  |  |

**Table S6. Spearman rank correlation coefficients between change in LARS score and changes in the FIQL scales and SF-12 scores**

|  | **FIQL lifestyle** | **FIQL coping** | **FIQL depression** | **FIQL embarrassment** | **SF-12 PCS** | **SF-12 MCS** |
| --- | --- | --- | --- | --- | --- | --- |
| **LARS score** | -0.39 | -0.39 | -0.05 | -0.23 | -0.09 | -0.22 |
| **FIQL lifestyle** |  | 0.39 | 0.39 | 0.50 | 0.36 | 0.26 |
| **FIQL coping** |  |  | 0.51 | 0.55 | 0.32 | 0.30 |
| **FIQL depression** |  |  |  | 0.53 | 0.21 | 0.38 |
| **FIQL embarrassment** |  |  |  |  | 0.36 | 0.33 |
| **Change in SF-12 PCS** |  |  |  |  |  | 0.06 |

The hue of the cell colour corresponds with the strength of the correlation.

The correlations between the LARS score and the separate domains of the FIQL and SF12 scores were <0.1 twice, approximately 0.2 twice and approximately 0.4 twice. This indicated that these other scales were collecting different (but complementary) information that the LARS score.
